# Supplementary material for: Wind turbines as new smokestacks: Preserving ruralness and restrictive land-use ordinances across U.S. counties
Source: PLoS One. 2023 Dec 13;18(12):e0294563. doi: 10.1371/journal.pone.0294563 (PMC10718419; doi:10.1371/journal.pone.0294563)
Supplement: Attachment — Submitted filename: Response to Reviewers county level restrictions.docx [file pone.0294563.s001.docx]

September 7, 2023

Baogui Xin, Ph.D.

Academic Editor

*PLOS ONE*

Subject: PONE-D-23-22939

Dear Dr. Xin,

Thank you for the opportunity to revise and resubmit our paper, “Wind turbines as new smokestacks: How the desire to preserve ruralness and limit landscape disruption motivates restrictive land-use ordinances across U.S. counties.” (previous title: “Are wind turbines the new smokestacks? Restrictive renewable energy land-use ordinances across U.S. counties, 2010-2022.”). We thank the reviewers for their thoughtful and constructive feedback. We are enclosing a response memo detailing the reviewers’ suggestions (*in italics*) and outlining ways we addressed them in the revised manuscript.

In your letter, you noted, “We require you to either (a) present written permission from the copyright holder to publish these figures specifically under the CC BY 4.0 license, or (b) remove the figures from your submission.” We believe this stipulation is not applicable to this paper because we have created Figures 1 and 2 ourselves based on the database we use in the manuscript. Regarding the information sources that have allowed us to construct our database, we have noted them in the paper’s text and also referenced them in the bibliography. These information sources are:

NCSL. (2021). State Renewable Portfolio Standards and Goals. <https://www.ncsl.org/energy/state-renewable-portfolio-standards-and-goals#:~:text=Renewable%20Portfolio%20Standards%20(RPS)%20require,production%20and%20encourage%20economic%20development> (Last accessed on June 12, 2023).

NREL. (2022). NREL Releases Comprehensive Databases of Local Ordinances for Siting Wind, Solar Energy Projects.<https://www.nrel.gov/news/program/2022/nrel-releases-comprehensive-databases-of-local-ordinances-for-siting-wind-solar-energy-projects.html> (Last accessed on March 6, 2023).

Finally, we have uploaded our replication materials to the Harvard Dataverse: <https://doi.org/10.7910/DVN/2VFK1V>.

Please let us know if you need anything else from us.

Sincerely,

Inhwan Ko, University of Nevada, Reno

Nives Dolšak, University of Washington, Seattle

Aseem Prakash, University of Washington, Seattle

**Response Memo**

Wind turbines as new smokestacks: How the desire to preserve ruralness and limit landscape disruption motivates restrictive land-use ordinances across U.S. counties

(previous title: Are wind turbines the new smokestacks? Restrictive renewable energy land-use ordinances across U.S. counties, 2010-2022)

PONE-D-23-22939

Dear Reviewers:

Thank you for your excellent and constructive feedback. We are enclosing a memo detailing your suggestions (*in italics*) and outlining specific ways we address them in the revised manuscript.

Sincerely,

Authors

**Reviewer 1**

1. *It is recommended to revise the abstract and add the innovation and contribution of this study in this section.*

Response:

Done.

2. *The format of this manuscript is not according to PLOS ONE requirements. Please revise it.*

Response:

Done.

3. *It is recommended to add a table to show the definitions of all the variables in the research in section 3.*

Response:

Done.

4. *Add a table to show the descriptive statistics of all variables in section 4*.

Response:

Done.

5. *As for the test of H2 and H3, I think that add interaction term or use subgroup regression is better.*

Response:

Thank you for this feedback. As we noted in the manuscript, a county’s ruralness is not a binary variable but a continuum. Therefore, we used the index of relative rurality. Creating subgroups would involve drawing an arbitrary distinction between rural and urban counties based on say population size, density, housing stock, or road connectivity. For example, if we take the cutoff as say 5,000 residents (as per the 2020 U.S. census), we will be placing both small urban areas that exist in the midst of rural areas, together with sprawling metropolis such as Los Angeles, whose residents might not view rural landscapes in their everyday lives. Hence, the continuum/index approach to assess ruralness is theoretically more suited to test our hypotheses.

6. *This study did not exclude the influence of other factors. For example, the authors said that renewable energy (RE) facilities provide a global public good of climate mitigation but impose local costs such as landscape disruption and harming the rural character, how about if the RE facilities do not impose local costs through technological advances or more perfect design. Please add some robust test*.

Response:

Good point. Arguably, in the future, there could be new technologies with smaller footprints that might not disrupt the rural landscape. Currently, however, we cannot think of any widely adopted RE technology that does not impose local costs. Might offshore wind projects fall in this category? As the controversy in New Jersey offshore wind reveals, local coastal communities, especially the fishing community, believe that offshore wind imposes local costs on them. In the concluding section, we acknowledge that with radical redesign and technical advances, the issue of landscape disruption might become less relevant.

7. *The empirical research surrounding this topic is too incomplete. H1: Counties with a higher installed capacity of RE facilities (solar and wind) are more likely to enact restrictive ordinances. This can be seen as an inevitable event to some extent. What is truly important in this research process is to study “why”. Please add the mechanism analysis to make the research more meaningful.*

Response:

Thank you. We have elaborated on the key mechanism, landscape disruption, in the revised paper.

**Reviewer 2**

1. *The variables and the empirical study cannot support the title. Or the author may choose to reconsider the title. As we can see in Section 3, the core explainable is the level of ruralness of the country, which cannot clearly proxy any critical factor concerning the title or the hypotheses. According to the hypotheses, there should be variables, which can stand for the restrictive ordinances. But we cannot find any. Also, the hypotheses are not closely related to the topic*.

Response:

Excellent suggestion. The new title is: “Wind turbines as new smokestacks: How the desire to preserve ruralness and limit landscape disruption motivates restrictive land-use ordinances across U.S. counties.”

2. *The logic of the paper is not clear, especially in Section 2. Hypothesis 2 and Hypothesis 3 cannot necessarily be drawn from their previous argumentation.*

Response:

Fair point, we have streamlined the argument.

3. *The conclusion section is too long as it seems to argue and deduce.*

Response:

We have shortened the conclusion.
