# Supplementary material for: Wind turbines as new smokestacks: Preserving ruralness and restrictive land-use ordinances across U.S. counties
Source: PLoS One. 2023 Dec 13;18(12):e0294563. doi: 10.1371/journal.pone.0294563 (PMC10718419; doi:10.1371/journal.pone.0294563)
Supplement: Attachment — Submitted filename: county level response_to_reviewers october 24.docx [file pone.0294563.s002.docx]

**Response Memo**

Wind turbines as new smokestacks:

Preserving ruralness and restrictive land-use ordinances across U.S. counties

(Previous title: Wind turbines as new smokestacks: How the desire to preserve ruralness and limit landscape disruption motivates restrictive land-use ordinances across U.S. counties)

PONE-D-23-22939R1

Dear Reviewers:

Thank you for your comments on how to revise the paper. We enclose a memo detailing your suggestions (*in italics*) and outline specific ways we address them in the revised manuscript.

Sincerely,

Authors

**Reviewer 2**

1. *The empirical section is sounder, but the logic is still not so clear. For example, how the ruralness is related to the existing level of wind and solar energy capacity?*

Response:

Thank you for the opportunity to refine our logic. Recent studies provide additional evidence that wind and solar energy facilities are more likely to be located in rural areas (Nilson and Stedman, 2022; O’Shaughnessy et al., 2023). The reason is that these facilities (especially solar) are land-intensive (Denholm et al., 2009; van de Ven et al., 2021), and rural areas have cheaper land. Meanwhile, the existing level of wind and solar energy capacity might increase the likelihood of the onset of land-use restrictions because as their negative impact becomes visible, existing facilities provoke local opposition (Bell et al., 2005; Gross, 2020). Therefore, we control for the existing level of wind and solar energy capacity because they might confound the relationship between the level of ruralness and the likelihood of land-use restrictions, the key variable of interest.

To refine the logic regarding Hypotheses 2 and 3, we have revised a paragraph on Page 7 as follows:

“Moreover, wind facilities tend to be larger than solar in terms of their generating capacity. In the U.S., the average capacity of wind energy facilities is 71.46MW, while that of solar energy facilities is 9.8 MW. Given that the mean capacity of wind turbines is about 2.75 MW, a single facility comprises 25 turbines, on average. It is therefore not surprising that wind facilities can often be visually spotted from a considerable distance, as opposed to solar facilities which are smaller in size and typically ground-mounted. Thus, in counties with more rural landscapes, the perceived landscape disruption of wind energy facilities may be larger than in counties without this characteristic. Therefore, rural counties will be more likely to introduce siting restrictions on wind energy facilities than urban counties. Because solar facilities are less visually disruptive in relation to wind energy facilities, we do not expect the level of ruralness to drive the onset of siting restrictions aimed at solar facilities.”

H2:	Rural counties are more likely to place siting restrictions on wind energy facilities than urban counties.

H3:	Rural counties are no different from urban counties in their likelihood of placing siting restrictions on solar energy facilities.

**Reference:**

Bell, D., Gray, T., & Haggett, C. (2005). The ‘social gap’ in wind farm siting decisions: explanations and policy responses. *Environmental Politics*, 14(4), 460-477.

Denholm P., Hand M., Jackson M, & Ong, S. (2009). Land-use requirements of modern wind power in the United States. National Renewable Energy Laboratory. Available from: <https://www.osti.gov/biblio/964608>

Gross, S. (2020). Renewables, land use, and local opposition in the United States. Brookings Institution. Available from:

<https://www.brookings.edu/articles/renewables-land-use-and-local-opposition-in-the-united-states/>

Nilson, R.S., and Richard C. Stedman (2022). Are big and small solar separate things?: The importance of scale in public support for solar energy development in upstate New York. *Energy Research & Social Science* 86: 102449.

O’Shaughnessy E., Wiser R., Hoen B., Rand J. & Elmallah S. (2023). Drivers and energy justice implications of renewable energy project siting in the United States. *Journal of Environmental Policy & Planning*, 25(3), pp.258-272.

Van de Ven D., Capellan-Peréz I., Arto I., Cazcarro I., de Castro C., Patel P. & Gonzalez-Eguino M. (2021). The potential land requirements and related land use change emissions of solar energy. *Scientific Reports*, 11(1), p.2907.

2. *Still the title is not so proper, not clear and very long.*

Response:

The new title is: “Wind turbines as new smokestacks: Preserving ruralness and restrictive land-use ordinances across U.S. counties.”
